# Metastatic renal cell carcinoma to the pancreas and subcutaneous tissue 10 years after radical nephrectomy: a case report

**DOI:** 10.1186/s13256-020-2355-6

**Published:** 2020-02-26

**Authors:** Wenjie Chin, Linping Cao, Xi Liu, Yufu Ye, Yuanxing Liu, Jun Yu, Shusen Zheng

**Affiliations:** 1 Division of Hepatobiliary and Pancreatic Surgery, Department of Surgery, First Affiliated Hospital, Zhejiang University School of Medicine, Hangzhou, 310003 China; 2 Key Lab of Combined Multi-Organ Transplantation, Ministry of Public Health, Hangzhou, 310003 China

**Keywords:** Renal cell carcinoma, Pancreas, Subcutaneous tissue, Metastasis, Synchronous

## Abstract

**Background:**

Synchronous renal cell carcinoma metastasizing to the pancreas and subcutaneous tissue is very rare. Unusual metastatic sites require attention during follow-up of renal cell carcinoma. It is extremely rare for renal cell carcinoma to metastasize to the pancreas; it is also very rare for it to metastasize to the subcutaneous tissue and extremely rare for it to synchronously metastasize to the pancreas and subcutaneous tissue almost a decade after radical nephrectomy. It is well known that most pancreatic tumors are primary pancreatic adenocarcinoma. However, the pancreas can also be an uncommon site for metastasis. We present a rare case of synchronous metastasis of renal cell carcinoma to the pancreas and subcutaneous tissue; we believe it to be only the second such case reported to date.

**Case presentation:**

We describe a case of a 74-year-old Chinese man who was diagnosed with metastatic renal cell carcinoma to the pancreas and subcutaneous tissue at the same time, 10 years after left radical nephrectomy. He received distal pancreatectomy with spleen preservation plus resection of the subcutaneous tissue lesions on the left side of the anterior abdominal wall and right waist. Pathology showed that all resected metastatic tumors were of the clear cell type. The patient was seen in regular follow-up afterward.

**Conclusion:**

Synchronous metastatic renal cell carcinoma to the pancreas and subcutaneous tissue is very rare, and it might occur after primary tumor resection. Patients must undergo lifelong monitoring and follow-up with regular examination so that any possible metastasis can be detected early. The optimal resection strategy should involve adequate resection margins and maximal tissue preservation of the pancreas, because renal cell carcinoma metastasizing to the pancreas and subcutaneous tissue has a good prognosis with long-term survival.

## Background

Renal cell carcinoma (RCC) is the most common type of renal tumor, accounting for about 2–3% of adult malignancies [1, 2]. It is reported that approximately 20–40% of patients will develop distant metastatic or locally recurring disease after radical nephrectomy [3]. The most frequent sites of metastasis are successively the lungs, lymph nodes, bones, liver, adrenal glands, and brain [4], whereas it is extremely rare for RCC to metastasize to the pancreas, and it is also very rare for RCC to metastasize to the subcutaneous tissue and extremely rare for RCC to synchronously metastasize to the pancreas and subcutaneous tissue after radical nephrectomy after almost a decade. It is well known that most pancreatic tumors are primary pancreatic adenocarcinoma. However, the pancreas can also be an uncommon site for metastasis. Pancreatic metastasis is rare, accounting for only 2–5% of pancreatic malignant tumors [5], and subcutaneous tissue metastatic clear cell RCC comprises 10% of all soft tissue metastasis [6]. We present a rare case of synchronous metastasis RCC to the pancreas and subcutaneous tissue that we believe is the second such case reported in the literature.

## Case presentation

**Fig. 1:**
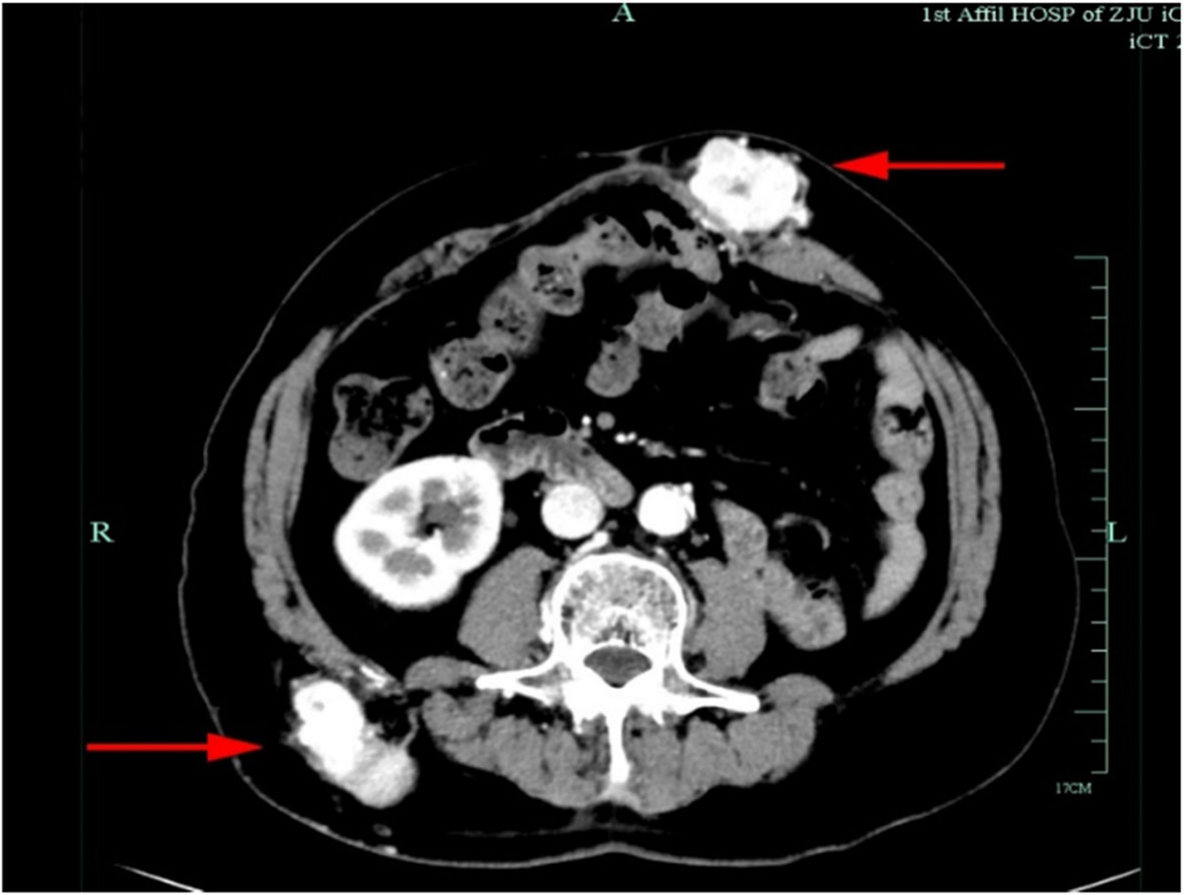
Enhanced computed tomographic scan showing a 5 × 6-cm mass in the left side of the anterior abdominal wall (*left arrow*) and a 5 × 6-cm mass in the back of the right waist (*right arrow*)

**Fig. 2:**
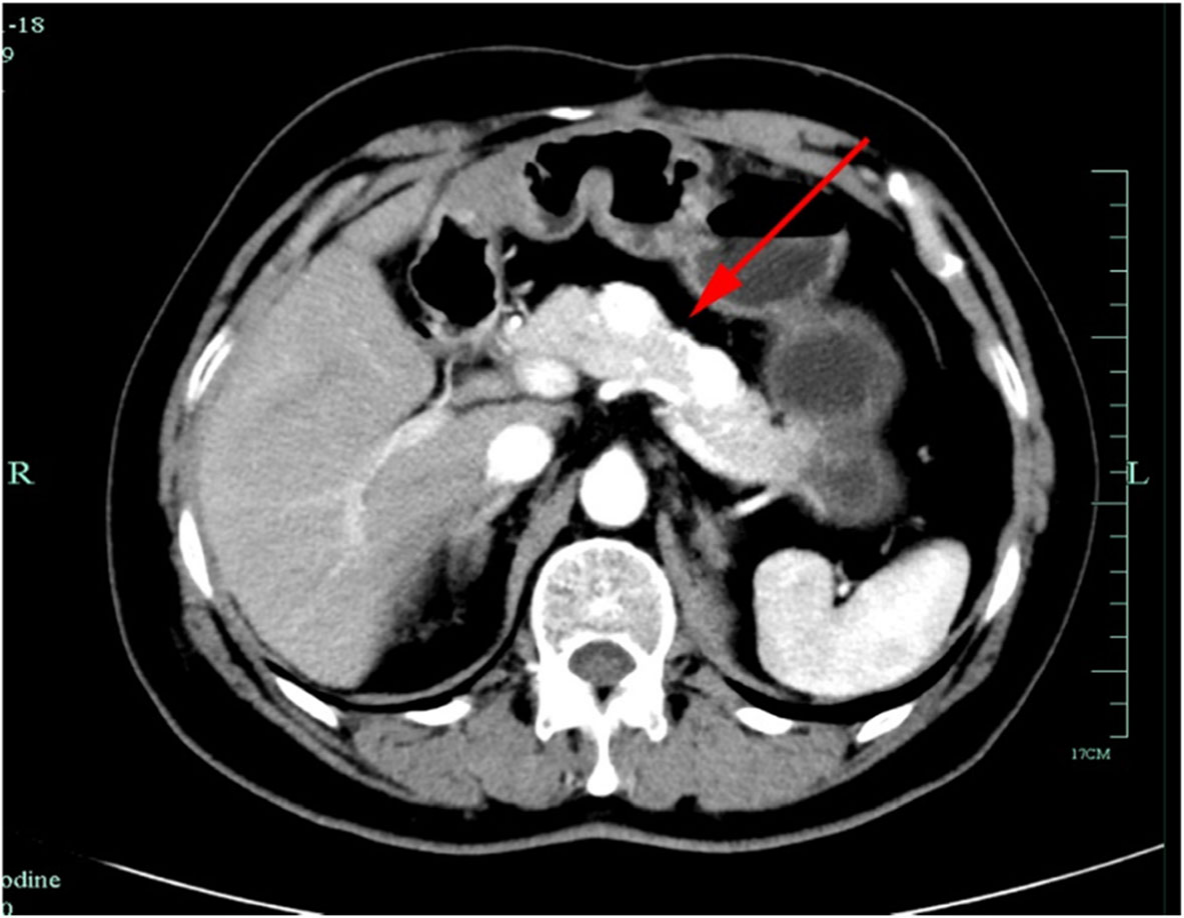
Enhanced computed tomographic scan showing a hypervascular lesion in the pancreas (*red arrow*)

Our patient was a 74-year-old Chinese man who had undergone left radical nephrectomy 10 years earlier. Postoperative pathological examination revealed clear cell carcinoma. One year later, he returned for laparoscopic cystectomy due to cholelithiasis, and we found a mass in the subcutaneous tissue protruding by about the size of a thumb into the abdomen. We did not resect the protruding mass, and we decided to see our patient on an annual follow-up basis. Nine years later, he came back to see us because he noticed the protruding mass in the subcutaneous tissue had grown larger within the last year. His physical examination revealed two masses, and enhanced computed tomography (CT) showed a 5 × 6-cm mass in the left side of the anterior abdominal wall and a 5 × 6-cm mass in the back of the right waist (Fig. 1). Enhanced CT also revealed a hypervascular lesion in the pancreas (Fig. 2). The patient’s tumor marker carcinoembryonic agent concentration was 6.0 ng/ml. Malignant tumors were suspected, and resection of the tumors was performed.

**Fig. 3:**
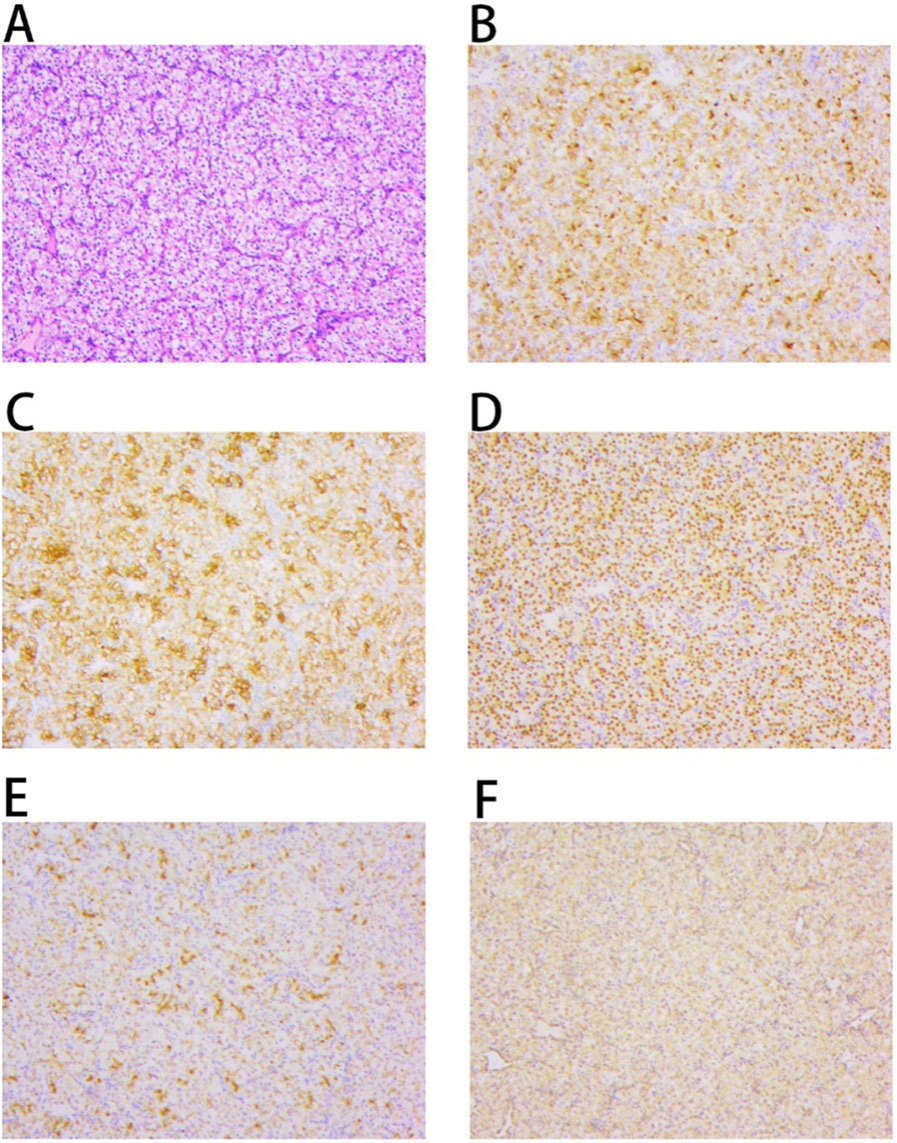
Histopathological examination of tissue samples. **a** Hematoxylin and eosin staining. **b** CD10^+^. **c** CK^+^. **d** PAX^+^. **e** RCC^+^. **f** Vimentin^+^. Original magnification, × 100. *CK* cytokeratin, *RCC* renal cell carcinoma

In surgery with the patient under general anesthesia, we first placed the patient in prone position to resect the tumor in the back of the right waist. Then, he was placed in supine position to resect the tumor in the left side of the anterior abdominal wall. Both tumors in the front and back were around 5 × 6 cm in size, and clear cell carcinoma was suspected. Later, we performed distal pancreatectomy with spleen preservation because enhanced CT showed a hypervascular lesion of approximately 3 × 3 cm in the pancreas. The size of the resected tumor in the left side of the anterior abdominal wall was 4 × 2.8 cm; the one in the right waist was 4 × 2.5 cm, and the ones from the pancreas were 1.8 × 1.3 cm and 1.9 × 1.5 cm. All resected tumors were of the clear cell type. Histopathological examination revealed they were paired box gene 8-positive (PAX8^+^), cluster of differentiation 10-positive (CD10^+^), RCC-positive, creatine kinase-positive (CK^+^), vimentin-positive, hepatocyte-negative, and thyroid transcription factor 1-negative (TTF-1^−^) (Fig. 3).

After surgery, our patient was seen in regular follow-up. One year later, our patient came back for a routine checkup, and CT showed recurrence in the pancreatic head. On the basis of our patient’s condition, our group offered him a palliative treatment plan, which is tyrosine kinase inhibitor (TKI) therapy. He refused any further treatment. The timeline of our patient’s case is listed in Table 1.

**Table 1:** Timeline of our patient’s case

Time	Event
2006	Left radical nephrectomy due to renal cell carcinoma
2007	Laparoscopic cystectomy due to cholelithiasis with finding of subcutaneous tissue mass
2007–2016	Follow-up of subcutaneous tissue mass in abdomen
2016	Subcutaneous tissue mass in abdomen enlarged
	Enhanced computed tomography (CT) revealing mass in left side of anterior abdominal wall and back of right waist, along with hypervascular lesion in the pancreas
	Surgical resection (distal pancreatectomy with spleen preservation plus subcutaneous tissue metastatic tumor resection)
2017	Routine checkup and CT revealing recurrence in the pancreatic head
	Patient refuses any further treatment
2017–2019	Follow-up

## Discussion

Synchronous metastasis to the pancreas and subcutaneous tissue from RCC is rare. In some clinical reports, the rate of RCC pancreatic metastasis ranges from 2% to 5% of malignant tumors [7–9], and subcutaneous tissue metastatic clear cell RCC comprised 10% of all soft tissue metastasis cases [6]. The pathological diagnosis of our patient’s case was relatively difficult because primary clear cell carcinoma in the pancreas and subcutaneous tissue is rare. To the best of our knowledge, we report the second case of synchronous metastasis to the pancreas and subcutaneous tissue from RCC.

RCC is well known for its different modes of presentation and its natural tendency to metastasize to many organs [10]. It can metastasize to the pancreas from RCC through a blood-borne route that involves parallel veins draining from the primary RCC lesion or through a lymphatic route whereby lymph passes the retroperitoneal nodes. Besides that, direct spreading to the pancreas from RCC is a rare optional route [11]. The metastatic pathway to the subcutaneous tissue remains to be elucidated.

The pancreatic and subcutaneous tissue metastasis of RCC lacks clinical characteristics. Most lesions are found during routine examination by ultrasound, CT scan, magnetic resonance imaging (MRI), positron emission tomography, and angiography [12], especially isolated pancreatic metastasis, whereas subcutaneous tissue metastasis can be found by palpation during physical examination because the patient will complain of discomfort or bulging of the mass. The most accurate procedure to evaluate the extent of metastasis is the CT scan. Hypervascular metastasis and nonfunctioning neuroendocrine tumor can also be differentiated using somatostatin receptor scintigraphy [12, 13].

The patient may be asymptomatic [14] if the metastatic lesion from RCC is tiny and isolated. In contrast, bigger tumors may cause discomfort, jaundice, and change in weight [15]. In our patient, the pancreatic metastatic nodules were found via enhanced CT, which showed hypervascular lesion characteristics. Surgical resection of metastatic disease to the pancreas and subcutaneous tissue is appropriate in certain clinical situations, depending on the virulence of the primary tumor, the spreading of metastatic disease, and the patient’s condition. The best efficacy for numerous pancreatic metastases from RCC can be achieved via complete surgical resection, which has a 5-year survival rate up to 75%. Those patients with obstruction in the pancreatic and biliary ducts can have relief after complete resection [16–18]. The specific method for surgical resection depends on the tumor’s location within the pancreas, mainly consisting of pancreaticoduodenectomy and middle segment or distal pancreatectomy [19]. The decision should be made to achieve clear margins of resection based on the tumor’s location within the pancreas [20]. If possible, avoid total pancreatectomy because complete tumor resection can be achieved with adequate resection margins and maximal tissue preservation [21]. Besides that, pancreatic resections performed in large healthcare facilities have low rates of mortality and morbidity [21].

Metastatic RCC overall has a poor result [10]; these patients’ survival may be improved using targeted drugs such as interferon immunotherapy after surgery [22]. CT scan is the most important diagnostic approach in preoperative decision-making. Hence, it is mandatory for patients to be monitored after nephrectomy because after decades of primary RCC, the pancreas can still be the locus for metastatic disease. Therefore, surgical removal of primary and metastatic tumors plus TKIs may be the best available treatment for these patients. Our patient underwent subcutaneous tissue resection and distal pancreatectomy with spleen preservation after left radical nephrectomy. Thus, complete surgical excision of the metastatic tumor may be the best available option for some patients [23].

## Conclusion

In conclusion, synchronous metastatic RCC to the pancreas and subcutaneous tissue is very rare, and it might occur several years after primary tumor resection. Therefore, patients with a history of RCC must be monitored and followed lifelong. A close follow-up scheme and regular examinations, including CT and MRI, are necessary so that any possible metastasis can be detected early. The optimal resection strategy should involve adequate resection margins and maximal tissue preservation of the pancreas because RCC metastasis to the pancreas and subcutaneous tissue has a good prognosis and long-term survival.

## Data Availability

Data sharing is not applicable to this article, because no datasets were generated or analyzed during the current study.
